# Comparative Physiological and Transcriptomic Analyses of Oat (*Avena sativa*) Seedlings under Salt Stress Reveal Salt Tolerance Mechanisms

**DOI:** 10.3390/plants13162238

**Published:** 2024-08-12

**Authors:** Xiangrui Zhou, Miaomiao Wang, Li Yang, Wenping Wang, Yuehua Zhang, Linbo Liu, Jikuan Chai, Huan Liu, Guiqin Zhao

**Affiliations:** 1 Gansu Provincial Key Laboratory of Aridland Crop Science, Lanzhou 730070, China; zhouxiangrui@gsau.edu.cn; 2 Key Laboratory of Forage Gerplasm Innovation and Variety Breeding of the Ministry of Agriculture and Rural Affairs, Key Laboratory of Grassland Ecosystem of the Ministry of Education, College of Grassland Science, Gansu Agricultural University, Lanzhou 730070, China; wangmm2321@163.com (M.W.); yangl071022@163.com (L.Y.); wangwp@gsau.edu.cn (W.W.); liulb93@163.com (L.L.); chaijk@gsau.edu.cn (J.C.); liuhuan@gsau.edu.cn (H.L.); 3 National Center of Pratacultural Technology Innovation (Under Preparation), Huhhot 010000, China; pp7768@126.com

**Keywords:** oat, salt stress, transcriptome, molecular mechanism, DEGs

## Abstract

Soil salinity is a major abiotic stress limiting crop production globally. Oat (*Avena sativa*) is an annual cereal with a strong salt tolerance, a high yield, and nutritional quality, although the mechanisms underlying its salt stress response remain largely unknown. We examined the physiological and transcriptomic responses of *A. sativa* seedlings to salt stress in tolerant cultivar Qingyongjiu 195 and sensitive cultivar 709. Under salt stress, Qingyongjiu 195 maintained a higher photosynthetic efficiency, antioxidant enzymes activity, and leaf K^+^ accumulation but a lower Na^+^ uptake than 709. RNA-seq revealed 6616 differentially expressed genes (DEGs), including 4265 up- and 2351 downregulated. These were enriched in pathways like plant–pathogen interaction, phenylpropanoid biosynthesis, and MAPK signaling. We specifically highlight DEGs involved in photosynthesis (*chlG*, *CP47 psbB*, *COX2*, *LHCB*) and antioxidants (*trxA*, *GroES*). Qingyongjiu 195 also appeared to enhance K^+^ uptake via *KAT1* and *AKT2* and sequester Na^+^ in vacuoles via *NHX2*. Additionally, *HKT* restricted Na^+^ while promoting K^+^ transport to shoots, maintaining K^+^/Na^+^. The expression levels of *CAX*, *ACA*, *CML*, *CaM*, and *CDPK* in Qingyongjiu 195 were higher than those in 709. Oats regulated Ca^2+^ concentration through CAX and ACA after salt stress, decoded Ca^2+^ signals through CML, and then transferred Ca^2+^ signals to downstream receptors through the Ca^2+^ sensors CaM and CDPK, thereby activating K^+^/Na^+^ transporters, such as SOS1 and NHX, etc. Our results shed light on plant salt stress response mechanisms and provide transcriptomic resources for molecular breeding in improving salt tolerance in oats.

## 1. Introduction

Soil salinity is a major abiotic stress limiting worldwide crop production [1]. Approximately 7% of the total land area (1 billion hectares) and 20% of the irrigated farmland in arid and semi-arid regions have been affected by salt, and this number continues to rise [2]. High salinity inhibits plant growth and development. Excessive Na^+^ absorption disrupts the cellular ionic balance, increasing reactive oxygen species (ROS) and secondary toxicity [3]. Salt stress also indirectly inhibits leaf photosynthesis, damages cell membranes and antioxidant defenses, and leads to the accumulation of organic osmolytes like proline and soluble sugars that mitigate stress [4]. Over time, plants have developed strategies for resisting salt damage. Exploiting saline-alkaline grasslands to screen for salt-tolerant forage varieties and elucidating their physiological and molecular stress resistance mechanisms can provide germplasm resources for breeding programs [5].

Oat (*Avena sativa*) is an annual cereal crop in the *Poaceae* family and an important food source in fragile ecological areas [6]. Oat production is concentrated in cold, high-altitude regions of North and Northwest China [7]. Oat possesses some saline-alkali tolerance, enabling cultivation across diverse soil types in semi-arid zones [8]. Recent studies on oat salt tolerance have focused on physiological and molecular responses during germination, seedling growth, and development. With increasing salt stress, the oat seed germination rate, germination potential, and vigor declined [9,10]. Leaf photosynthetic pigments, net photosynthesis, intercellular CO_2_, transpiration, stomatal conductance, and other photosynthetic indicators generally decreased, with greater declines in salt-sensitive versus tolerant cultivars [11]. Numerous studies found that antioxidant enzymes and osmolytes were higher in tolerant cultivars, and low salinity had little effect on antioxidants [12,13,14]. Under salt stress, oat seedlings selectively absorbed and transported K^+^ and Na^+^, with roots storing more Na^+^ and stems accumulating K^+^ [15]. The calcium signaling pathway is linked to the activation of K^+^/Na^+^ transporters, such as SOS1 and NHX. Due to the large number of Ca^2+^ transport and binding proteins involved in calcium signal transduction and the complex interaction between these proteins, there are many unsolved mysteries in the calcium signal network [16]. The first step in solving these problems is to identify the genes involved.

High-throughput transcriptome sequencing now enables the identification and screening of key transcripts and the correlation of physiological processes underlying plant salt stress responses. For example, RNA-seq revealed differentially expressed genes involved in oxidative stress and redox reactions in rice [17] and bluegrass [18], calcium signaling, oxidative phosphorylation, secondary metabolite synthesis, ROS clearance, and ion homeostasis in wheat [19], *Leymus mollis* [20], and maize [21] under salt stress, but there may be relatively little research specific to oats. With recent advances in oat genome sequencing [22], RNA-seq has become a valuable tool for elucidating the molecular mechanisms of salt tolerance in this crop.

Therefore, in this study, we aimed to elucidate the mechanisms underlying oat responses to salt stress using transcriptomic approaches. The results revealed physiological and molecular responses of different salt-resistant oat varieties to salt stress, with a focus on DEGs related to photosynthesis, antioxidant systems, and ion transport. The transcriptome expression profiles of oat under salt stress obtained in this study provide important clues for further research on the mechanism of salt tolerance in oat. Moreover, the differentially expressed genes under salt stress identified in this study may be highly beneficial for identifying suitable genes for biotechnological manipulation to improve salt tolerance in oat.

## 2. Results and Analysis

### 2.1. Physiological Changes under Salt Stress

**Figure 1:**
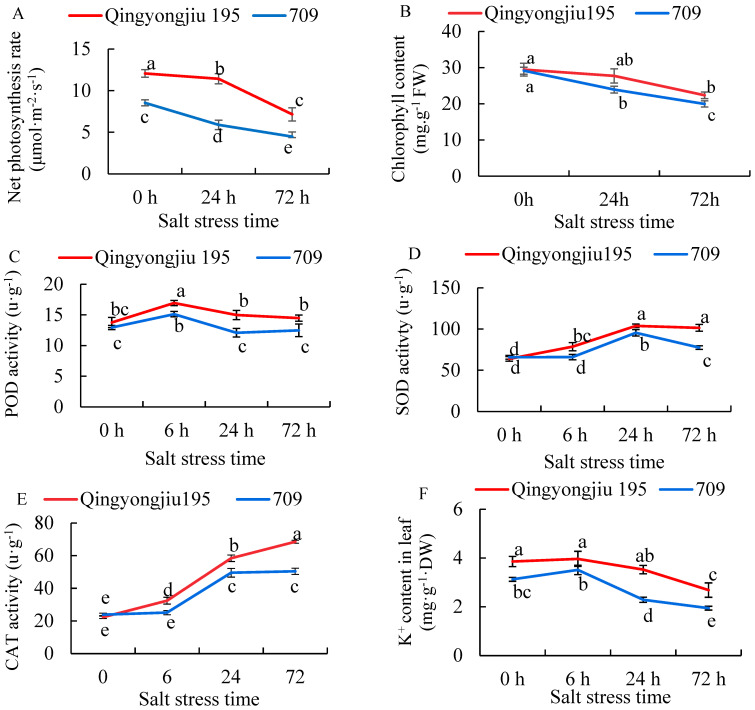

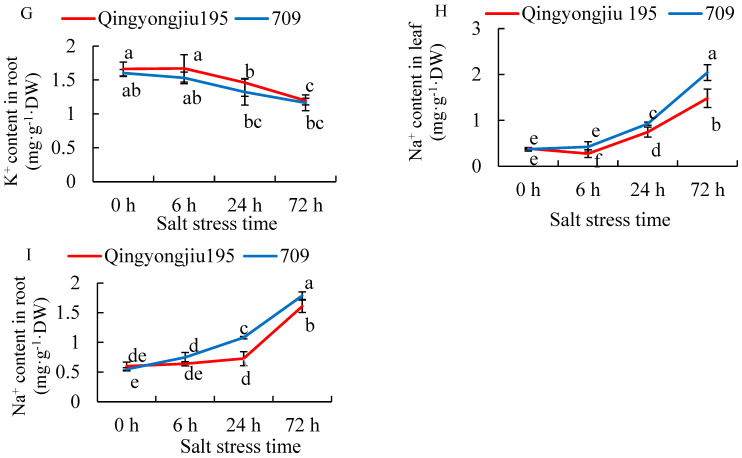
Physiological changes in oat seedlings treated with 0.9% NaCl at different times. (**A**) Net photosynthesis rate; (**B**) Chlorophyll content; (**C**) POD activity; (**D**) SOD activity; (**E**) CAT activity; (**F**) Leaf K^+^ content; (**G**) Root K^+^ content; (**H**) Leaf Na^+^ content; (**I**) Root Na^+^ content. Note: Different lowercase letters indicate significant differences among different times and between two varieties at *p* < 0.05. This applies to all subsequent figures.

The salt-tolerant Qingyongjiu 195 exhibited a significantly higher net photosynthetic rate than the salt-sensitive 709 under normal conditions (Figure 1A). Salt stress reduced the net photosynthetic rate in both cultivars, but the decline was greater in 709 than in Qingyongjiu 195, especially at 24 h (Figure 1A). After 24 and 72 h of salt stress, the net photosynthetic rate in Qingyongjiu 195 decreased by 5.34% and 40.74%, while that in 709 decreased by 31.03% and 47.58%, respectively (Figure 1A). Salt stress also decreased the leaf chlorophyll content in both cultivars, but the decline was less pronounced in Qingyongjiu 195 than in 709 (Figure 1B). At 24 and 72 h of stress, the respective leaf chlorophyll content of Qingyongjiu 195 was still 15.8% and 12.1% higher than that of 709 (Figure 1B).

Prolonged salt stress increased the activities of antioxidant enzymes, which were generally more significant in Qingyongjiu 195 than in 709. Peroxidase (POD) activity first increased then decreased over time, peaking at 6 h after stress with 23.11% and 16.76% increases over 0 h in Qingyongjiu 195 and 709, respectively. No significant differences occurred among 0, 24, and 72 h (Figure 1C). The superoxide dismutase (SOD) activity peaked at 24 h after stress, increasing by 62.43% in Qingyongjiu 195 and by 44.92% in 709 over 0 h (Figure 1D). The catalase (CAT) activity increased over time and was higher in Qingyongjiu 195 than in 709. At 6, 24, and 72 h of salt stress, the CAT activity increased by 45.10%, 161.52%, and 207.67%, respectively, over 0 h in Qingyongjiu 195 and by 5.19%, 107.58%, and 111.31% in 709 (Figure 1E).

The leaf and root K^+^ content decreased while the Na^+^ content increased over time in both cultivars under salt stress. The leaf K^+^ was higher in Qingyongjiu 195 than in 709 at 0, 6, 24, and 72 h, by 23.3%, 12.9%, 54.2%, and 38%, respectively (Figure 1F). No significant difference occurred in the root K^+^ between the cultivars (Figure 1G). The leaf Na^+^ was lower in Qingyongjiu 195 than in 709 at 6, 24, and 72 h, by 53.0%, 24.2%, and 37.7%, respectively (Figure 1H). The root Na^+^ was also lower in Qingyongjiu 195 than in 709, by 48.6% and 11.2% at 24 and 72 h, respectively (Figure 1I).

### 2.2. Quality Analysis of De Novo Assembly and Sequencing

Transcriptomic sequencing generated 251.57 Gb of data across salt-tolerant Qingyongjiu 195 and salt-sensitive 709 after 0, 6, 24, and 72 h of salt treatment (Table S2). The total raw reads per sample ranged between 64.27 and 9101 Mb. After filtering, 55.67–76.04 Mb clean reads were obtained per sample. All samples had >8.35 Gb clean bases with a >84% Q30 ratio and 48.32–49.68% GC content, indicating high-quality sequencing suitable for downstream analysis.

### 2.3. Unigene Functional Annotation and Classification

Unigenes were compared against NR (Non-Redundant Protein Sequence Database), NT (Nucleotide Sequence Database), SwissProt, KEGG (Kyoto Encyclopedia of Genes and Genomes), KOG (clusters of euKaryotic Orthologous Groups), Pfam (Protein family database), and GO (Gene Ontology) databases for annotation (Table S3). In total, 209,226 unigenes were annotated, with 142,095 (67.91%) in NR, 126,868 (60.64%) in NT, 102,633 (49.05%) in SwissProt, 104,683 (50.03%) in KEGG, 111,240 (53.17%) in KOG, 105,715 (50.53%) in Pfam, and 74,687 (35.70%) in GO. Of these, 46,305 unigenes (22.13%) were co-annotated in all databases, while 154,328 (73.76%) were annotated in at least one database. NR annotation showed the highest homology to *Aegilops tauschii* subsp. tauschii (32.71%), followed by *Brachypodium distachyon* (23.03%), *Hordeum vulgare* (11.69%), *Aegilops tauschii* (6.32%), and *Triticum urartu* (5.79%) (Figure S1).

### 2.4. Analysis of Differentially Expressed Genes (DEGs)

Figure S2 showed that salt stress altered the expression of many genes in both cultivars, with more upregulated genes in Qingyongjiu 195 than in 709 at 6 and 72 h. The number of up-regulated DEGs was 23,858 in 195_0 vs. 709_0, 16,834 in 195_6 vs. 709_6, 24,980 in 195_24 vs. 709_24, and 15,259 in 195_72 vs. 709_72. The downregulated genes numbered 13,012 in 195_0 vs. 709_0, 17,479 in 195_6 vs. 709_6, 21,423 in 195_24 vs. 709_24, and 12,946 in 195_72 vs. 709_72.

**Figure 2:**
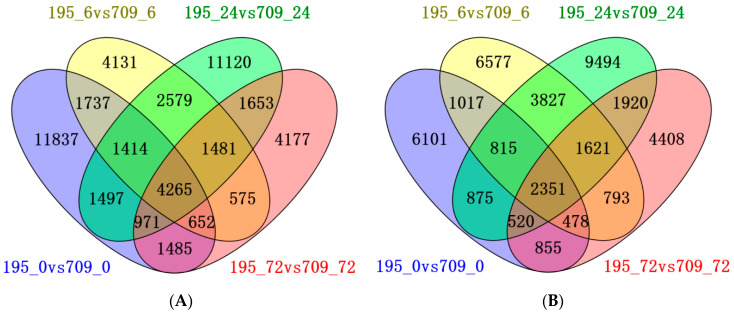
Venn diagram of DEGs between Qingyongjiu 195 and 709 at different salt stress times: (**A**) overlapping upregulated DEGs in four comparison groups: 195_0 vs. 709_0 (blue), 195_6 vs. 709_6 (yellow), 195_24 vs. 709_24 (green), and 195_72 vs. 709_72 (pink); (**B**) overlapping downregulated DEGs in these four comparison groups. 195_0, 195_6, 195_24, and 195_72 represent the salt-tolerant variety Qingyongjiu 195 treated with 0.9% NaCl at 0, 6, 24, and 72 h, respectively. 709_0, 709_6, 709_24, and 709_72 represent the salt-sensitive variety 709 treated with 0.9% NaCl at 0, 6, 24, and 72 h, respectively. For example, 195_6-vs-709_6 represents the comparison of the number of DEGs between Qingyongjiu 195 and 709 treated with 0.9% NaCl for 6 h. This labeling applies to subsequent figures.

We compared the gene expression profiles of the two cultivars at four time points: 195_0 vs. 709_0, 195_6 vs. 709_6, 195_24 vs. 709_24, and 195_72 vs. 709_72. We identified 4265 overlapping upregulated genes (Figure 2A) and 2351 overlapping downregulated genes (Figure 2B) among the four comparison groups.

### 2.5. GO Analysis of DEGs

**Figure 3:**
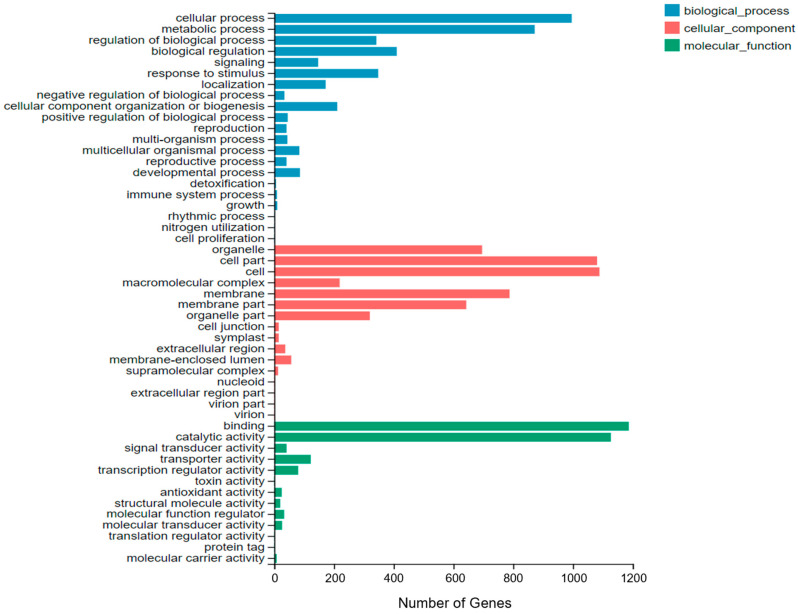
Genes ontology (GO) classifications of DEGs in the four comparison groups (195_0 vs. 709_0, 195_6 vs. 709_6, 195_24 vs. 709_24, and 195_72 vs. 709_72). The results are summarized in three main GO categories: biological process, cellular component, and molecular function.

We analyzed the overlapping differentially expressed genes between Qingyongjiu 195 and 709 under the same salt stress duration. Figure 3 shows that 6616 genes from the four comparison groups (i.e., 195_0 vs. 709_0, 195_6 vs. 709_6, 195_24 vs. 709_24, and 195_72 vs. 709_72) were enriched in 50 GO secondary categories. These genes were mainly involved in cellular processes, metabolic processes, biological regulation, and responses to stimuli, with 996, 872, 342, and 410 enriched genes, respectively. In the cellular component category, the genes were predominantly enriched in the cell, cell part, and membrane, with 1089, 1081, and 788 enriched genes, respectively. In the molecular function category, the genes were mostly enriched in binding, catalytic activity, and transporter activity, with 1187, 1127, and 122 enriched genes, respectively.

### 2.6. KEGG Enrichment of DEGs

**Figure 4:**
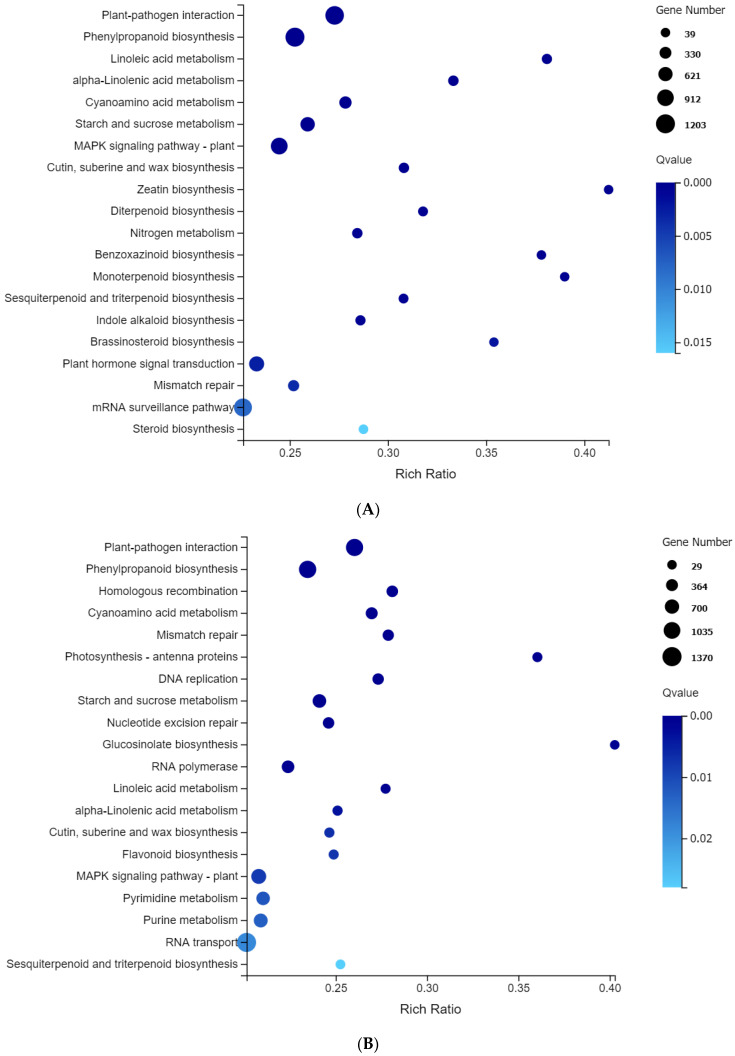

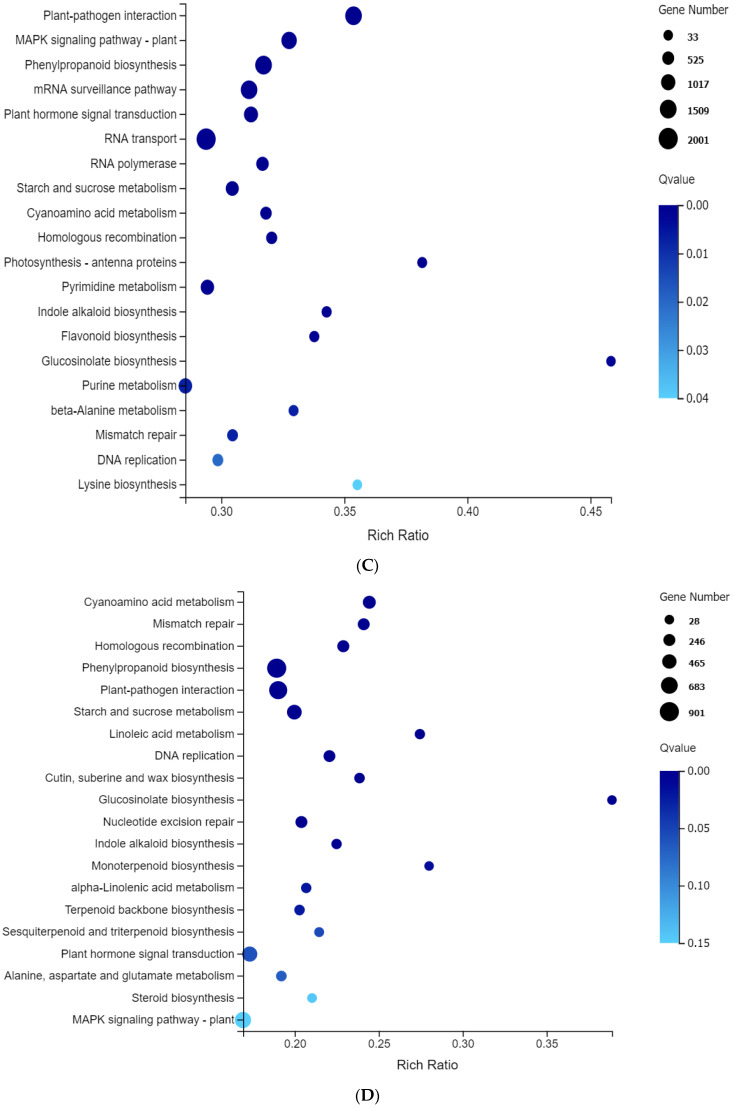
Summary statistics for Kyoto Encyclopedia of Genes and Genomes (KEGG) pathway enrichment of differential expression genes. The vertical axis shows the pathway, and the horizontal axis shows the enrichment factor. The size of the dot represents the number of DEGs in the pathway. The dot color corresponds to the range of Q-values: (**A**) 195_0 vs. 709_0; (**B**) 195_6 vs. 709_6; (**C**) 195_24 vs. 709_24; and (**D**) 195_72 vs. 709_72.

KEGG enrichment analysis was performed on the differentially expressed genes (DEGs) of two oat varieties subjected to salt stress for the same duration. Four comparison groups, 195_0 vs. 709_0 (Figure 4A), 195_6 vs. 709_6 (Figure 4B), 195_24 vs. 709_24 (Figure 4C), and 195_72 vs. 709_72 (Figure 4D), were all enriched in 132 metabolic pathways. Plant–pathogen interaction, phenylpropanoid biosynthesis, cyanoamino acid metabolism, and starch and sucrose metabolism were significantly enriched in each comparison group. In addition, we observed that linoleic acid metabolism pathways, MAPK signaling pathways, and plant hormone signal transduction pathways were significantly enriched at 0 h (Figure 4A). At 6 h, linoleic acid metabolism pathways, the MAPK signaling pathway, and photosynthesis–antenna proteins were enriched. At 24 h, the MAPK signaling pathway, plant hormone signal transduction pathways, and photosynthesis–antenna proteins were significantly enriched. At 72 h, linoleic acid metabolism was enriched.

### 2.7. Analysis of DEGs in Oat under Salt Stress

#### 2.7.1. Analysis of DEGs Related to Photosynthesis

**Figure 5:**
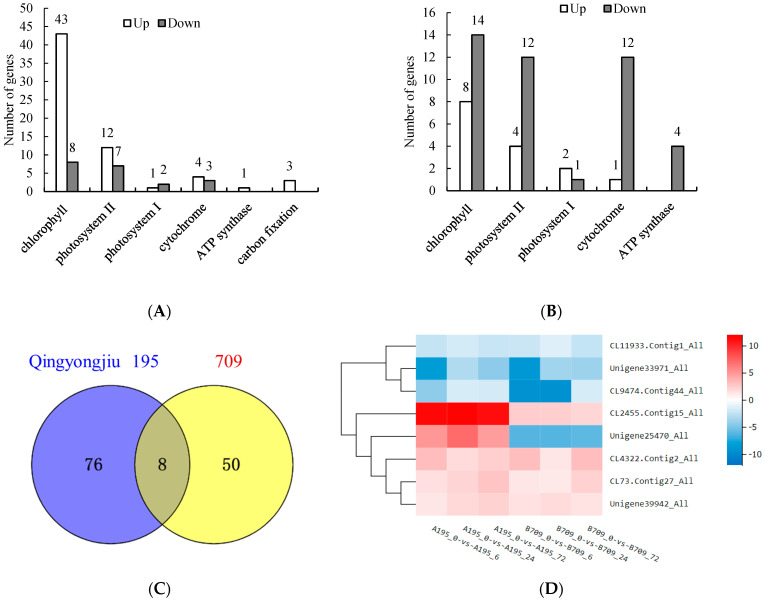
Number of DEGs related to photosynthesis in Qingyongjiu 195 (**A**) and 709 (**B**), Venn diagrams of DEGs in Qingyongjiu 195 and 709 (**C**), and cluster heat map of DEGs (**D**). The chlorophyll biosynthesis gene *chlG* (CL11933.Contig1_All), photosynthetic electron transport-associated gene in PS I (CL2455.Contig15_All), chloroplast-associated gene (CL4322.Contig2_All), photosystem II CP47 psbB chlorophyll protein gene (CL9474.Contig44_All), COX2 cytochrome c oxidase gene (Unigene25470_All), and chlorophyll a-b binding protein LHCB genes (CL73.Contig27_All, Unigene33971_All, Unigene39942_All).

Since the photosynthesis of higher plants is a photosynthetic electron transport system composed of chlorophyll, photosystem II (PS II), photosystem I (PS I), cytochrome, ATP synthase, and carbon fixation, the expression of differentially expressed genes (DEGs) related to these components in Oat treated with 0.9% NaCl was analyzed. A total of 84 DEGs were identified in Qingyongjiu 195 at the three time points of stress, among which 43, 12, 1, 4, 1, and 3 genes related to chlorophyll, PS II, PS I, cytochrome, ATP synthase, and carbon fixation were upregulated, respectively; at the same time, there were eight, seven, two, and three downregulated genes related to chlorophyll, PS II, PS I, and cytochrome, respectively (Figure 5A). Most of the DEGs related to chlorophyll, PS II, and cytochrome, as well as all the DEGs related to ATP synthase and carbon fixation, were upregulated, indicating that these genes were responsible for the normal photosynthesis in Qingyongjiu 195 under salt stress.

In contrast, 709 had 58 DEGs at the three time points of stress, among which eight, four, two, and one genes related to chlorophyll, PS II, PS I, and cytochrome were upregulated, respectively; at the same time, there were 14, 12, 1, 12, and 4 downregulated genes related to chlorophyll, PS II, PS I, cytochrome, and ATP synthase, respectively (Figure 5B). Most of the DEGs related to chlorophyll, PS II, PS I, and cytochrome and all of the DEGs related to ATP synthase were downregulated, indicating that the expression of the photosynthesis-related genes was inhibited in 709 under salt stress.

The Venn diagram of DEGs at the three time points of salt stress in Qingyongjiu 195 and 709 revealed that eight genes were differentially expressed between the two varieties (Figure 5C). These genes included the chlorophyll biosynthesis gene *chlG* (CL11933.Contig1_All), a photosynthetic electron transport-associated gene in PS I (CL2455.Contig15_All), a chloroplast-associated gene (CL4322.Contig2_All), the photosystem II CP47 psbB chlorophyll protein gene (CL9474.Contig44_All), the COX2 cytochrome c oxidase gene (Unigene25470_All), and the chlorophyll a-b binding protein LHCB genes (CL73.Contig27_All, Unigene33971_All, and Unigene39942_All).

Theifferrential gene cluster heat map (Figure 5D) showed that the expression levels of these genes varied at different periods under salt stress, but the expression trend was similar. It is noteworthy that the expression of the cytochrome c oxidase COX2 gene (Unigene25470_All) was completely different between the two varieties. *COX2* was upregulated at the three time points in Qingyongjiu 195 but downregulated in 709. Additionally, the expression of the photosynthetic electron transport-associated gene in PS I, CL2455.Contig15_All, was much higher in Qingyongjiu 195 than in 709 6 h, 24 h, and 72 h after salt treatment.

#### 2.7.2. Analysis of DEGs Associated with Reactive Oxygen Species (ROS) Scavenging Systems

**Figure 6:**
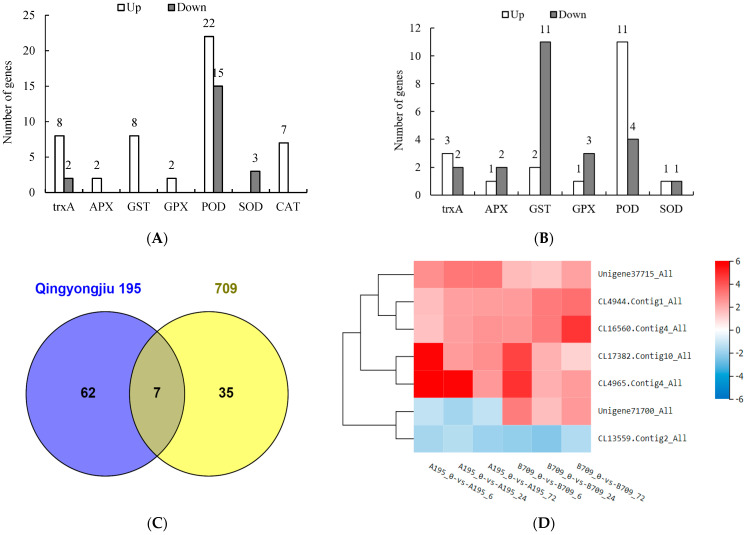
Number of DEGs related to the reactive oxygen species (ROS) scavenging system in Qingyongjiu 195 (**A**) and 709 (**B**), Venn diagrams of DEGs in Qingyongjiu 195 and 709 (**C**), and cluster heat map of DEGs (**D**). The chaperone GroES gene (CL13559.Contig2_All), which regulates SOD activity, and the POD genes (Unigene37715_All, CL4944.Contig1_All, CL16560.Contig4_All, CL17382.Contig10_All, CL4965.Contig4_All, Unigene71700_All).

The reactive oxygen species (ROS) clearance system in higher plants is primarily composed of the ascorbate-glutathione (AsA-GSH) cycle, glutathione peroxidase (GPX) pathway, catalase (CAT) pathway, and peroxidase/thioredoxin (PrxR/Trx) pathway. The differentially expressed genes (DEGs) related to these pathways were analyzed in Qingyongjiu 195 and 709 after salt stress. As shown in Figure 6A,B, a total of 69 DEGs related to the ROS clearance system were identified in Qingyongjiu 195 after salt stress for 6, 24, and 72 h, with 8 thioredoxin (trxA) genes upregulated and 2 downregulated, 22 peroxidase coding genes (POD) upregulated and 15 downregulated, 3 superoxide dismutase (SOD) genes downregulated, and ascorbate peroxidase (APX), glutathione S-transferase (GST), GPX, and CAT genes upregulated. These findings suggest that these genes play a crucial role in the salt tolerance of Qingyongjiu 195. In contrast, a total of 42 DEGs related to the ROS clearance system were identified in 709 after 6, 24, and 72 h of salt stress. With the exception of a greater number of upregulated genes in POD compared to downregulated genes, most DEGs related to the ROS clearance system pathway were downregulated (Figure 6B), attributed to the sensitivity of 709 to salt stress.

As can be observed from the Venn diagram (Figure 6C), there are seven DEGs in Qingyongjiu 195 and 709. These genes comprise the chaperone GroES gene (CL13559.Contig2_All), which regulates SOD activity, and the POD genes (CL16560.Contig4_All, CL17382.Contig10_All, CL4944.Contig1_All, CL4965.Contig4_All, Unigene37715_All, and Unigene71700_All). According to the cluster heat map (Figure 6D), the expression of these seven genes was upregulated after salt stress in both varieties, and the expression pattern was similar. Although Unigene71700_All was upregulated in 709 and downregulated in Qingyongjiu 195, the expression ratio of other POD genes in Qingyongjiu 195 was higher than that in 709, indicating the importance of the POD genes in the salt-resistant material Qingyongjiu 195.

#### 2.7.3. Analysis of Differentially Expressed Genes Related to Na^+^ and K^+^ Transport

Maintaining Na^+^/K^+^ homeostasis is critical for plant salt tolerance. We thus analyzed the expression profiles of major Na^+^ and K^+^ transporter genes in Qingyongjiu 195 and 709 after 0.9% NaCl treatment for 6, 24, and 72 h.

As shown in Table 1, a total of eight oat genes related to Na^+^/K^+^ transport were differentially expressed under salt stress. These included genes encoding the vacuolar membrane Na^+^/H^+^ antiporters NHX2 (CL6997.Contig13_All) and NHX6 (CL4133.Contig1_All), potassium channels AKT2 (CL17722.Contig19_All) and KAT1 (CL3919.Contig1_All), potassium ion transporter HAK18 (CL18436.Contig3_All), plasma membrane Na^+^/H^+^ antiporter SOS1 (CL4014.Contig17_All), and high-affinity potassium transporters HKT (CL2008.Contig3_All) and HKT3 (CL6904.Contig3_All).

**Figure 7:**
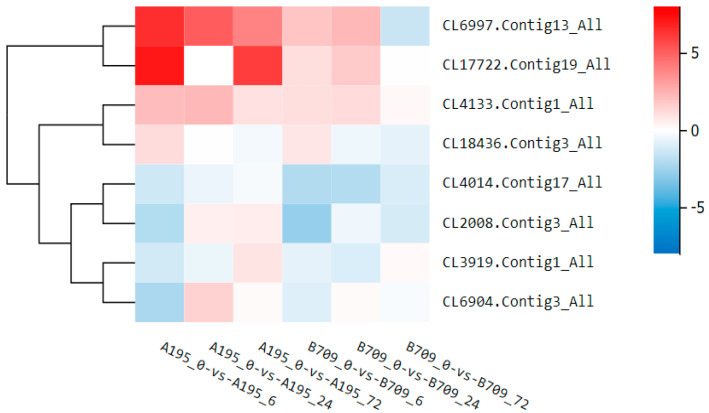
Cluster heat map related to Na^+^, K^+^ transport under salt stress. Vacuolar membrane Na^+^/H^+^ antiporters NHX2 (CL6997.Contig13_All); NHX6 (CL4133.Contig1_All), potassium channels AKT2 (CL17722.Contig19_All) and KAT1 (CL3919.Contig1_All), potassium ion transporter HAK18 (CL18436.Contig3_All), plasma membrane Na^+^/H^+^ antiporter SOS1 (CL4014.Contig17_All), and high-affinity potassium transporters HKT (CL2008.Contig3_All) and HKT3 (CL6904.Contig3_All).

As shown in Figure 7, *NHX6* and *SOS1* were differentially upregulated or downregulated, respectively, across all time points in both varieties. *KAT1* displayed opposite regulation at 6, 24, and 72 h. *AKT2* expression increased at 6 h in Qingyongjiu 195 but varied over time in 709. The expression pattern of *NHX2* diverged between the two varieties after 72 h of stress. *HKT6* was upregulated later in Qingyongjiu 195 but downregulated in 709, whereas *HKT3* upregulation occurred later in Qingyongjiu 195 only. These findings indicate the variety-specific tuning of Na^+^/K^+^ transporter expression conferring improved salt tolerance.

#### 2.7.4. Analysis of Expressed Genes Related to Ca^2+^ Transport

**Figure 8:**
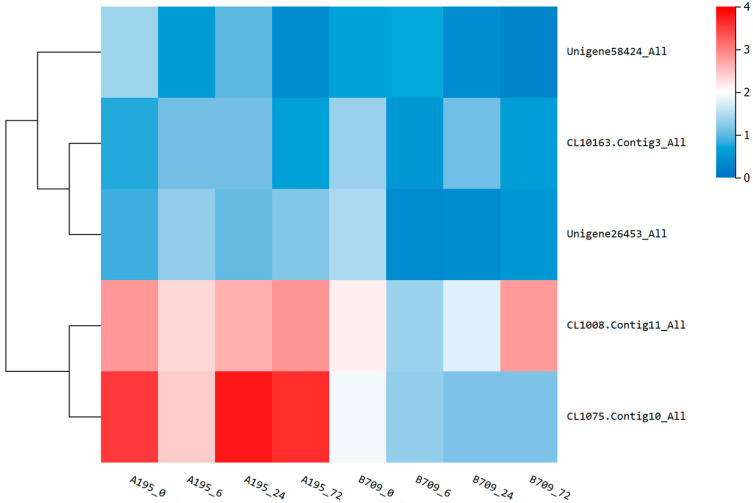
Cluster heat map related to Ca^2+^ transport under salt stress. Ca^2+^-transporting ATPase ACA (Unigene58424_All), Ca^2+^-binding protein CML (CL10163.Contig3_All), Ca^2+^/H^+^ antiporter CAX (Unigene26453_All), Ca^2+^-dependent protein kinase CDPK (CL1008.Contig11_All), Calmodulin CaM (CL1075.Contig10_All).

Ca^2+^ has long been recognized as a conserved second messenger and plays a vital role in plant responses to various environmental stimuli, including salinity and drought stress. Therefore, we found some calcium transport-related genes (Ca^2+^-transporting ATPase ACA, Ca^2+^-binding protein CML, Ca^2+^/H^+^ antiporter CAX, Ca^2+^-dependent protein kinase CDPK, Calmodulin CaM) from the transcriptome database and analyzed their expression in the two varieties. As shown in Figure 8, the expression levels of these five genes in Qingyongjiu 195 were higher than those in 709. The expression of ACA in Qingyongjiu 195 decreased with the increase in time; however, the expression levels of CML, CAX, CDPK, and CaM in Qingyongjiu 195 reached the highest at 24 h, 6 h, 72 h, and 24 h, respectively. In addition, these five genes were divided into two groups according to function: ACA, CML, and CAX as one group and CDPK and CaM as one group. The expressions of CDPK and CaM were higher than those of ACA, CML, and CAX. These findings suggest that calcium plays an important role in the salt tolerance of oat.

### 2.8. Fluorescence Quantitative PCR Analysis

To validate the reliability of RNA-Seq results, 22 differentially expressed genes related to photosynthesis, antioxidant activity, and Na^+^/K^+^ transport from Qingyongjiu 195 and 709 under 6, 24, and 72 h salt stress were selected for qRT-PCR experiments.

For Qingyongjiu 195, the expression trends of the 22 DEGs showed strong consistency with RNA-seq data, with correlation coefficients of 0.99625, 0.88519, and 0.88683 at 6, 24, and 72 h, respectively (Figure S3A,C,E). Similarly, the expression patterns of the 22 DEGs in 709 were highly concordant with the RNA-seq results, exhibiting correlation coefficients of 0.8128, 0.8735, and 0.8802 at the corresponding timepoints (Figure S3B,D,F).

These results demonstrate that qRT-PCR analysis validated the differential expression observed via RNA-Seq, corroborating the reliability of the transcriptome profiling for investigating gene regulation under salt stress in the two oat varieties.

## 3. Discussion

Plant responses to salt stress are highly complex, involving interactions between physiological processes, metabolic pathways, and molecular and cellular regulation [23]. While oat salt tolerance has been studied at different levels, the underlying mechanisms remain poorly understood.

RNA-seq represents a valuable tool for obtaining a nearly complete characterization of transcriptomic events occurring at a specific stage. Gene annotation is an important step in data analysis in which biological information is attached to predicted genes or unigenes. The presence of a high proportion of unigenes with a high similarity to protein sequences from other plant species helps confirm the integrity of transcript sequences assembled from sequencing data [24]. In the current study, 67.91% of unigenes matched at least one homolog in the NR database, as determined by BLAST and functional bioinformatics analyses, which helps confirm the reliability of the assembled oat unigenes and suggests that these sequences can be used for further investigations.

In this study, differentially expressed genes (DEGs) in Qingyongjiu 195 and 709 increased rapidly after 0, 6, 24, and 72 h of 0.9% NaCl treatment and then decreased, indicating that oat induces many genes early under short-term stress. Over time, fewer DEGs occurred in Qingyongjiu 195 versus 709. Qingyongjiu 195 also exhibited more upregulated DEGs at 6 and 72 h, reflecting its complexity in salt responses.

The statistics of common DEGs at each timepoint identified 4268 shared upregulated genes in Qingyongjiu 195 but only 2724 in 709. This suggests that a greater ability to activate protective genes under stress contributes to salt tolerance in Qingyongjiu 195. Previous studies have reported that salt stress is associated with metabolic processes, cellular processes, stimulus responses, cellular components, and binding and catalytic activities in plants [25,26]. Relevant pathways include photosynthesis, hormone signaling, phenylpropane biosynthesis, and MAPK signaling [27].

Our KEGG analysis revealed enriched pathways in Qingyongjiu 195 alone, such as photosynthesis–antenna proteins, photosynthesis, carbon fixation, porphyrin/chlorophyll metabolism, and amino acid metabolism. These differences in signaling, transcription, and secondary metabolism likely underlie variations in salt tolerance between oat varieties. Collectively, our results provide new insights into oat salt tolerance mechanisms warranting further exploration.

Photosynthesis is vital for plant growth and development, as it converts light energy to chemical energy through electron transport, providing ATP for carbon assimilation [28]. Key genes regulate chlorophyll synthesis, photosystem II (PSII) and I (PSI), cytochromes, ATP synthase, and carbon fixation pathways under salt stress [29].

In this study, salt treatment decreased the photosynthetic rate and chlorophyll content over time for both oats, albeit less severely in Qingyongjiu 195 versus 709. This suggests salt induces photosynthetic genes, enhancing efficiency and reducing damage in Qingyongjiu 195.

RNA-seq identified 84 and 58 salt-responsive photosynthetic genes in Qingyongjiu 195 and 709, respectively. In Qingyongjiu 195, most DEGs related to chlorophyll, PSII, cytochromes and ATP synthase/carbon fixation DEGs were upregulated. However, in 709, most DEGs for these functions were downregulated. Among them, 43 genes related to chlorophyll were upregulated and 8 were downregulated in Qingyongjiu 195, while 8 were upregulated and 14 were downregulated in 709. This may be the reason why the chlorophyll content of Qingyongjiu 195 is higher than that of 709; it matched the chlorophyll measurement results.

Potentially enhancing light energy conversion and ATP production, DEGs in Qingyongjiu 195’s electron transport chain may confer stronger salt tolerance through maintained photosynthesis. Co-expression analysis identified eight variably expressed genes between varieties. Cytochrome c oxidase COX2 (Unigene25470_All) upregulation persisted in Qingyongjiu 195 but not in 709. The photosynthetic electron transport-associated gene in PS I (CL2455.Contig15_All) was upregulated in both, albeit more strongly in Qingyongjiu 195 due to possible inhibition in 709.

In summary, oat appears to be able to regulate photosynthetic efficiency under salt stress by promoting chlorophyll synthesis, electron transport, and cytochrome transcription.

Salt stress causes metabolic imbalances and increased reactive oxygen species (ROS) production, leading to oxidative stress that damages lipids, DNA, and proteins [30]. This study found that under prolonged salt treatment, peroxide (POD) activity peaked at 6 h, superoxide dismutase (SOD) peaked at 24 h, and catalase (CAT) increased over time in both oats. However, antioxidant enzyme activities were consistently higher in Qingyongjiu 195 versus 709, indicating an ability to maintain defenses early in stress. Prolonged stress may cause higher ROS in oats, inhibiting SOD and POD synthesis but increasing CAT to alleviate damage, consistent with the observation in Wu et al. [31]. Lu and Li [32] found that many peroxisome, ascorbate, and glutathione pathway genes were upregulated in salt-tolerant *Elytrigia repens*. Here, 69 and 42 ROS-scavenging differentially expressed genes (DEGs) were detected in Qingyongjiu 195 and 709 after stress, respectively, with most upregulated only in Qingyongjiu 195. This stronger ROS-clearance system likely confers its greater salt tolerance. Key plant pathways remove ROS/H_2_O_2_ via ascorbate-glutathione, glutathione peroxidase, catalase, and peroxiredoxin/thioredoxin systems [33]. In oat, stress induces ascorbate peroxidase, glutathione S-transferase, glutathione peroxidase, and catalase upregulation. A total of 8 thioredoxin trxA genes and 22 peroxidase genes were also upregulated strongly in Qingyongjiu 195. In addition, this study also found that a total of seven genes were differentially expressed in Qingyongjiu 195 and 709, including one *GroES* gene, which regulate SOD activity, and six POD genes, and their expressions were upregulated after salt stress, but the upregulation factor of most genes in Qingyongjiu 195 was greater than that of 709. The expression or activation of these genes or pathways may be the main factor for the difference in antioxidant enzyme activity between the two varieties. 

Collectively, these genes stress-induced antioxidant cooperation, with higher upregulation in Qingyongjiu 195, suggesting critical roles in ROS scavenging and oat salt tolerance. In addition, some studies have shown that the sensing of salinity stress at the plasma membrane activates *RBOH* genes and generates reactive Oxygen Species (ROS) at the apoplast. ROS generated from the organelles chloroplast, peroxisome, and mitochondria trigger cellular oxidative burst [34,35]. ROS and calcium activate MAPK genes and the downstream transcription factors NAC and bZIP in bread wheat. MAPK signaling induces cellular antioxidant and compatible osmolyte biosynthesis and imparts tissue tolerance to salinity [36]. Therefore, the relationship between the MAPK pathway, calcium signal, and antioxidant properties, as well as the identification and functional analysis of related genes, will be an important part of the study of oat salt tolerance mechanisms.

Maintaining K^+^/Na^+^ homeostasis is critical for plant survival under salt stress. ROS bursts mediate the triggering of putative RCDs and calcium signaling improves sodium exclusion and vacuolar sodium compartmentation by activating SOS1, VP1, and NHX1 [37,38]. This study found higher K^+^ content but lower Na^+^ in Qingyongjiu 195 leaves versus 709, indicating an ability to preferentially retain K^+^ aboveground during stress. Ion transporters regulate ion distributions. Vacuolar *NHX* antiporters sequester excess cytoplasmic Na^+^ into vacuoles [39], as seen by *NHX6* upregulation across stresses in both oats. *SOS1* downregulation likely enhanced Na^+^ efflux [37]. *NHX2*’s divergent expression at 72 h, upregulated only in Qingyongjiu 195, suggests a key role in its stronger tolerance [40]. *KAT1* expression followed similar time-dependent patterns as stress progressed but was induced to a greater degree in Qingyongjiu 195 versus 709. *AKT2* upregulation under prolonged stress also occurred exclusively in Qingyongjiu 195. Preferential K^+^ absorption and transport via these transporters likely contributed to ion balance in Qingyongjiu 195. As high-affinity Na^+^ transporters or co-transporters with K^+^, differential *HKT* upregulation in Qingyongjiu 195 indicates the better maintenance of intracellular K^+^/Na^+^ homeostasis through the preferential transport of K^+^ aboveground and the restriction of Na^+^ accumulation [41]. 

Many external signals including changes in K^+^/Na^+^ status in the soil are sensed by plants through calcium signaling mechanisms [42]. It has long been proposed that Ca^2+^/H^+^ exchangers (CAXs, antiporter) and Ca^2+^-ATPases (ACAs) are responsible for transferring Ca^2+^ from the cytosol to either external space or intracellular stores [43]. In parallel, the Ca^2+^- binding proteins (CML) constitute an integral part of the toolkit for Ca^2+^ sensing and decoding processes [44]. To translate immune receptor-induced Ca^2+^ elevations into immunity programs, Ca^2+^-binding proteins are first in line to decode the Ca^2+^ signals. These proteins exhibit varying affinity to Ca^2+^, which allows them to turn on and off in response to changing Ca^2+^ concentrations [45,46]. Ca^2+^-binding causes conformational changes in these Ca^2+^ sensors, which often initiate functional interactions with downstream effectors [47]. Previous studies have primarily centered on two types of Ca^2+^ sensors, calmodulin (CaM) and Ca^2+^-dependent protein kinases (CDPKs), which serve as decoders that bridge Ca^2+^ elevations to immune responses [48,49]. CDPKs are the best-characterized calcium sensors and have a unique structure which can transfer Ca^2+^ signals via phosphorylation events [50] and participate in plant development and responses to environmental stress [51,52]. In this study, we identified these genes respectively and found that their expression in the salt-tolerant variety Qingyongjiu 195 was significantly higher than that of the salt-sensitive variety 709, and the expression levels of CaM and CDPK were higher than those of other genes. These results revealed that oats regulated the Ca^2+^ concentration through CAX and ACA after salt stress, decoded Ca^2+^ signals through CML, and then transferred Ca^2+^ signals to downstream receptors through the Ca^2+^ sensors CaM and CDPK, thereby activating K^+^/Na^+^ transporters, such as SOS1 and NHX, etc. However, gene families such as CML, CDPK, and CAX have many members, and the specific function of each member is not the same, which needs to be further studied. 

## 4. Materials and Methods

### 4.1. Experimental Materials

The experimental materials were two husked oat cultivars, salt-tolerant Qingyongjiu 195 and salt-sensitive 709. Qingyongjiu 195 was previously identified as salt-tolerant based on comprehensive early-stage evaluation [53]. Salt-sensitive 709 was identified through the salinity screening of oat germplasm by our research group. The experiments were conducted in 2021 at the College of Grassland Science laboratory, Gansu Agricultural University.

### 4.2. Experimental Treatments

Full, healthy seeds were selected, soaked in 75% ethanol for 1 min to disinfect, rinsed with distilled water, and sown in 9 cm diameter × 13 cm height seedling cups filled with sand. The cups were placed in trays in a growth chamber with a 16 h photoperiod, day/night temperatures of 25 ± 1 °C/18 ± 1 °C, and 55% relative humidity. The seedlings were watered daily with distilled water before germination. At the two true leaf stage, 1 L Hoagland nutrient solution was applied every 2 days. Based on the germination pre-trials, 0.9% NaCl was selected for salt treatment [54] and added to the nutrient solution after 3 weeks of growth. Leaves were collected at 0, 6, 24, and 72 h after the salt treatment onset, frozen in liquid nitrogen, and stored at −80 °C for physiological and transcriptomic analyses. Each treatment was replicated three times.

### 4.3. Physiological Parameters Determination

Catalase (CAT) activity was determined by the ultraviolet absorption method; superoxide dismutase (SOD) activity was determined by the nitro blue tetrazolium colorimetry method; peroxidase (POD) activity was determined by the guaiacol colorimetry method [55].

From 9:00 to 11:00 am, the net photosynthesis rate (Pn) of leaves in oat was measured by an automatic photosynthetic measuring apparatus (GFS-3000, Heinz Walz GmbH, Effeltrich, Germany). The chlorophyll content was determined by the acetone extraction method [56].

The contents of K^+^ and Na^+^ of leaves and roots in oat were determined by a flame photometer [57].

### 4.4. Transcriptomic Analysis

#### 4.4.1. RNA Extraction, cDNA Library Construction, and RNA-Seq

The total RNA from oat leaves was extracted by Trizol reagent (Invitrogen, Carlsbad, CA, USA). The quality and integrity of the RNA samples were determined via Agarose gel electrophoresis in the Agilent 2100 Bioanalyzer (Agilent Technologies, Santa Clara, CA, USA). The extracted RNA samples were reverse-transcribed using the first strand cDNA synthesis kit (TIANGEN, Beijing, China) in a 20 μL volume. The specific components and volumes of the system are as follows: 4 μL 5 × FastKing-RT SuperMix, 5 μL Total RNA, 11 μL RNase-free ddH_2_O. The reverse transcription reaction was performed at 42 °C for 15 min and at 95 °C for 3 min. Sequencing adaptors were linked to the purified cDNA, and 24 double-strand libraries were obtained by PCR amplification. Transcriptome sequencing was completed using the BGISEQ-500 platform by Beijing Genomics Institution (BGI, Beijing, China). The raw sequence data have been deposited in the Genome Sequence Archive (CRA004020) at the Beijing Institute of Genomics (BIG) Data Center, Chinese Academy of Sciences. Available online: http://bigd.big.ac.cn/gsa (accessed on 5 January 2023).

#### 4.4.2. De Novo Transcriptome Assembly and Unigene Functional Annotation

The raw image data obtained by sequencing were converted into raw data (raw data or raw reads) by base calling, and then the raw reads were filtered. That is, reads containing joints (joint contamination), low-quality reads, and reads with unknown base information of more than 5% were removed, and then clean reads were obtained. Trinity was used for the de novo assembly of clean reads, Tgicl was used to cluster the assembled transcripts to remove redundancy, and the final Unigenes were obtained. Clean reads were aligned to reference gene sequences using Bowtie2. The assembled Unigenes were used as query sequences against the KEGG; GO; NR; NT; Swiss-Prot; Pfam; KOG; and TF databases.

#### 4.4.3. Identification and Analysis of Differentially Expressed Genes (DEGs)

The expression level of each unigene was calculated and normalized as Fragments Per Kilobase Million (FPKM) values using RSEM (v1.2.8) software. The identification of DEGs was based on the negative binomial distribution of the DEseq2 package. The cut-off for DEGs was a fold change ≥1 and an adjusted *p* value ≤ 0.001. 

### 4.5. qRT-PCR Analysis

A total of 22 candidate DEGs involved in photosynthesis, antioxidant properties, and K^+^/Na^+^ transport were selected for qRT-PCR validation. Specific primer pairs for the selected genes were designed and are listed in Table S1. The cDNA was transcribed from 5 μg total RNA using the SuperScript II system (Invitrogen, Shanghai, China) according to the manufacturer’s instructions. The qRT-PCR was carried out with SYBR Premix Ex-Taq (TaKaRa, Dalian, China) on an ABI QuantStudio 7 Flex RT-PCR instrument (Applied Biosystems, Waltham, MA, USA), with reaction volumes of 20 μL that contained 10 μL 2 × SuperRealPreMix Plus, 1 μL Forward primer, 1 μL Reverse primer, 5 μL cDNA, and 3 μL RNase-free ddH_2_O. The relative expression level of the selected genes was presented as the fold-change calculated using the 2^−ΔΔCt^ method. 

### 4.6. Statistical Analysis

The data of the physiological parameters were collected by Microsoft Excel 2016. The mean value and standard error were calculated to conduct variance analysis for each processing character of the tested oat varieties. Statistical analyses were performed using a one-way ANOVA followed by Duncan’s Multiple Range Tests (DMRT) in SPSS version 25.0.

## 5. Conclusions

This study elucidated that the salt-tolerant variety Qingyongjiu 195 possesses stronger salt tolerance than the sensitive variety 709. This is primarily due to Qingyongjiu 195’s elevated photosynthetic characteristics (chlorophyll content and net photosynthetic rate) and antioxidant enzyme activity (SOD and POD during initial stress stages, and CAT during later stages) combined with a high K^+^ maintenance while limiting Na^+^ transport aboveground. 

RNA-seq identified 6616 differentially expressed genes (DEGs; 4265 upregulated, 2351 downregulated) mainly involved in plant–pathogen interaction, phenylpropanoid biosynthesis, linoleic acid metabolism, MAPK signaling pathways, cyanoamino acid metabolism, and starch and sucrose metabolism.

Expression profiles elucidated potential salt tolerance regulatory mechanisms in Qingyongjiu 195. Compared with the salt-sensitive variety 709, more chlorophyll-related genes and antioxidase-related genes were upregulated in the salt-tolerant variety Qingyongjiu 195. This is the main factor that causes Qingyongjiu 195 to have higher photosynthetic efficiency and antioxidant enzyme activity. Its ability to enhance photosynthetic efficiency involves regulating the chlorophyll biosynthesis genes *chlG* and *CP47 psbB*, as well as the cytochrome c oxidase *COX2* and light-harvesting complex *LHCB*. Stronger antioxidant defense may stem from regulating thioredoxin *trxA* and *GroES*. Additionally, Qingyongjiu 195 can regulate cellular K^+^ uptake via *KAT1* and *AKT2* transporters and sequester excess Na^+^ into vacuoles using *NHX2*. *HKT* transporters likely restrict Na^+^ influx while promoting K^+^ transport aboveground to maintain ion balance. Oats regulated the Ca^2+^ concentration through CAX and ACA after salt stress, decoded Ca^2+^ signals through CML, and then transferred Ca^2+^ signals to downstream receptors through the Ca^2+^ sensors CaM and CDPK, thereby activating K^+^/Na^+^ transporters, such as the SOS pathway and NHX, etc.

This study provides novel insights into oat salt stress mechanisms, shedding light on salt-responsive genes amenable to future molecular breeding efforts aimed at developing more salt-tolerant crops.

## Figures and Tables

**Table 1:** Genes related to Na^+^, K^+^ transport under salt stress.

Gene ID	Log_2_(Fold Change)		Qingyongjiu 195	Log_2_(Fold Change)		709	Gene
	6 h/0 h	24 h/0 h	72 h/0 h	6 h/0 h	24 h/0 h	72 h/0 h	
CL6997.Contig13_All	6.43	5.08	3.92	1.86	2.26	−1.48	NHX2
CL17722.Contig19_All	7.12	-	6.04	1.07	1.69	−0.04	AKT2
CL4133.Contig1_All	2.14	2.24	0.97	1.08	1.15	0.23	NHX6
CL18436.Contig3_All	1.14	0.02	−0.32	0.81	−0.46	−0.70	HAK18
CL4014.Contig17_All	−1.32	−0.55	−0.25	−2.05	−2.01	−1.06	SOS1
CL2008.Contig3_All	−2.03	0.63	0.51	−2.76	−0.44	−1.14	HKT6
CL3919.Contig1_All	−1.24	−0.52	0.89	−0.71	−1.02	0.18	KAT1
CL6904.Contig3_All	−2.24	1.44	0.15	−0.92	0.16	−0.21	HKT3

## Data Availability

Data is contained within the article and Supplementary Materials.

## Supplementary Materials

The following supporting information can be downloaded at: https://www.mdpi.com/article/10.3390/plants13162238/s1.
