# Supplementary material for: Comparative Physiological and Transcriptomic Analyses of Oat (Avena sativa) Seedlings under Salt Stress Reveal Salt Tolerance Mechanisms
Source: Plants (Basel). 2024 Aug 12;13(16):2238. doi: 10.3390/plants13162238 (PMC11359270; doi:10.3390/plants13162238)
Supplement: Supplementary file 1 [file plants-13-02238-s001.zip › plants-3108889-supplementary.pdf]

Supplementary Materials

Table S1 Genes and its primers for qRT-PCR

Gene ID	Forward primer	Reverse primer
CL73.Contig27_All	AAGCAGGTTTCCTCAGGCAG	TGTTCTTGGCGAAGGTCTCG
CL4965.Contig4_All	GACGGATGTCGTGGTCTTGT	CCCTTCTTGGTCAGCAGGTT
CL9474.Contig46_All	CAGCGATCTGGCATTGGGTA	CCCGTTACATGAAATGCGCC
Unigene33971_All	TATCTGTGCGTGCCTCGTG	TAGTCGTACGGGATGGGACC
CL4944.Contig1_All	TCGGACAACATCATGCCGAG	TGTAGAGGCGGAACATGACG
CL11933.Contig1_All	CGGTTGCGCGCTCGCCTGGT	CATCAATGTCTCGATCGTACC
CL2455.Contig15_All	CGCCCAACAATGGTGCATGC	CGACGCCACCACGTCGATG
CL4322.Contig2_All	CACCGAGCTAACTCTCGTG	GGCTGCACGCGGTCTGCC
Unigene25470_All	GAGCTTATCACCTTTCATG	CTACGGTCAATGCTCTG
Unigene39942_All	GGCTCCAGCAGTCCATGGT	CTGGCTGCGTGATCTGAGAC
CL13559.Contig2_All	CGGCGTACTTGGAGTACAG	GCCTACCGCGGCACCGCAG
CL16560.Contig4_All	TGCCAACCAGCAGCGCAATG	ATGGCCTCCCGCACGATCTC
CL17382.Contig10_All	ACCTCACTACCAGCCCGCTC	GCGATCTTCTCGCTGTCGG
Unigene37715_All	AGCCGATGGTGTGCGCTCCG	CGTAGTAGCCGACCCTGAGCT
Unigene71700_All	CGGCAGTTGATCCTGACTTCC	CGCCTGCGCCATCGCGGAC
CL18436.Contig3_All	GAAGGCGTAGACATAGTCG	TCTCGACGGCCAATGACAG
CL2008.Contig3_All	TGCTGGATATGAAGCTCTGAAG	GTTGTGAGCTTCATCATTAT
CL3919.Contig1_All	TGTAGCTTATGTCGACAGC	TCCATGGAGTGTCCAACAGT
CL4014.Contig17_All	GCACGGCCTCGGCAAGCTCG	TGATTGCTTACATTGCGAAC
CL4133.Contig1_All	TGCTGTTCTTACATAGCTAC	CATCGTTCACCGCCCTTGAC
CL6904.Contig3_All	CTCTCGGTGACACATACAAGC	TACAGTGATCGATATCGAG
CL6997.Contig13_All	GAGTTGTGTGCAGCTCTC	TCACCCTCGGTGCCATGGC
Actin	GAGCGGGAAATTGTGAGGGA	GAAGAGGACCTCAGGGCAAC

Table S2 Statistics of the sequencing data quality

Sample	Total Raw Reads (M)	Total Clean Reads (M)	Total Clean Bases(Gb)	Clean Reads Q30(%)	GC(%)
195_0_1	87.84	73.70	11.06	85.12	48.52
195_0_2	75.26	63.36	9.50	84.87	49.19
195_0_3	83.71	70.44	10.57	85.30	49.15
195_6_1	87.69	73.08	10.96	85.90	48.91
195_6_2	84.40	70.74	10.61	86.18	48.76
195_6_3	69.11	59.45	8.92	86.15	48.86
195_24_1	89.60	76.04	11.41	85.32	48.52
195_24_2	73.19	63.34	9.50	85.69	49.11
195_24_3	89.60	75.97	11.40	86.19	48.66
195_72_1	76.29	66.06	9.91	85.78	49.36
195_72_2	89.59	74.36	11.15	84.49	48.93
195_72_3	81.80	69.64	10.45	85.22	49.66
709_0_1	85.06	72.77	10.91	86.45	48.36
709_0_2	84.33	72.17	10.83	86.02	49.15
709_0_3	86.08	74.33	11.15	85.30	48.41
709_6_1	87.84	72.86	10.93	84.91	48.68
709_6_2	87.84	73.8	11.07	84.85	48.58
709_6_3	87.84	73.29	10.99	84.53	48.87
709_24_1	88.38	75.55	11.33	84.23	48.32
709_24_2	87.84	74.10	11.11	85.23	48.39
709_24_3	91.01	74.91	11.24	85.04	49.07
709_72_1	76.01	64.75	9.71	85.63	49.24
709_72_2	66.71	56.76	8.51	85.11	49.68
709_72_3	64.27	55.67	8.35	85.82	49.64

Table S3 Unigene functional annotation according to the major database

Database	Number of genes	Percentage (%)
NR	142,095	67.91
NT	126,868	60.64
Swissprot	102,633	49.05
KEGG	111,240	53.17
KOG	104,683	50.03
Pfam	105,715	50.53
GO	74,687	35.70
Intersection	46,305	22.13
Overall	154,328	73.76
Total	209,226	100

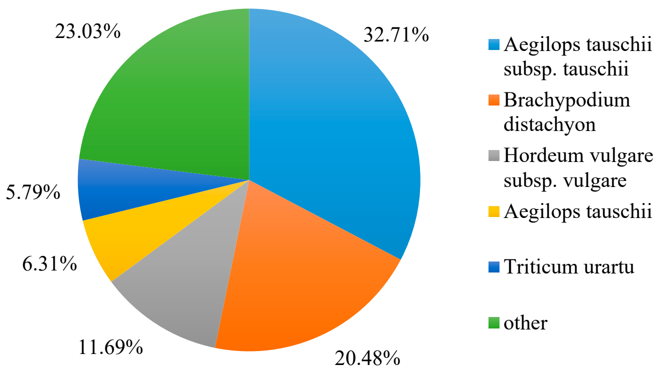

Figure S1 Homologous plant species classification

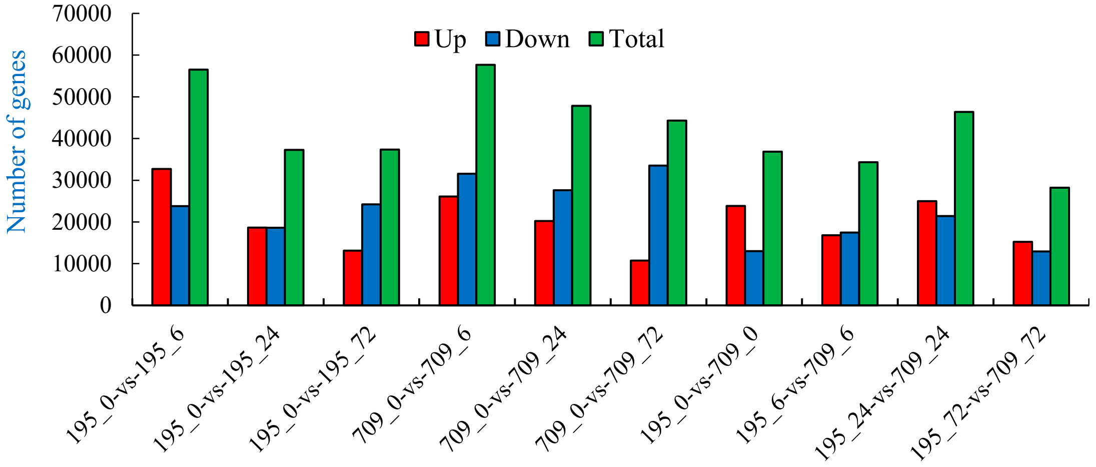

Figure S2. Number of up-regulated, down-regulated, and total DEGs in 10 comparisons. 195_0, 195_6, 195_24, and 195_72 represent salt-tolerant variety Qingyongjiu 195 treated with 0.9% NaCl at 0, 6, 24, and 72 h, respectively. 709_0, 709_6, 709_24, and 709_72 represent salt-sensitive variety 709 treated with 0.9% NaCl at 0, 6, 24, and 72 h, respectively. For example, 195_0-vs-195_6 represents the comparison of the number of DEGs between Qingyongjiu 195 treated with 0.9% NaCl for 6 hours and 0 hours. This labeling applies to subsequent figures.

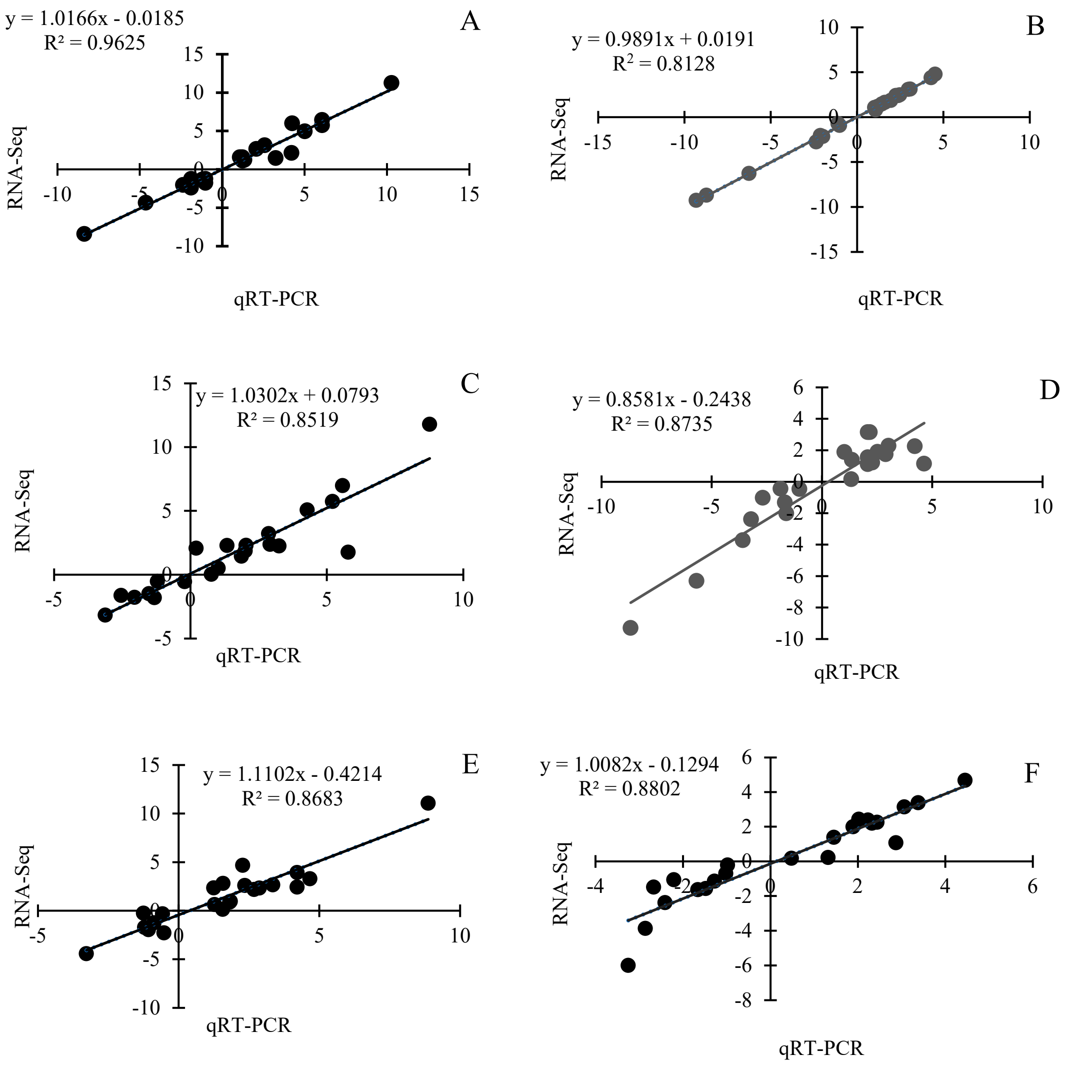

Figure S3 Correlation of expression analysis by RNA-seq (X-axis) and qRT-PCR (Y-axis) in Qingyongjiu 195 and 709 under salt stress at 6h (A, B), 24h (C, D) and 72h (E, F), respectively
